# Supplementary material for: Local connectome phenotypes predict social, health, and cognitive factors
Source: Netw Neurosci. 2018 Mar 1;2(1):86–105. doi: 10.1162/NETN_a_00031 (PMC5989992; doi:10.1162/NETN_a_00031)
Supplement: Supplementary file 1 [file netn-02-86-s001.pdf]

Powell, M. A., Garcia, J. O., Yeh, F.-C., Vettel, J. M., & Verstynen, T. (2017). Supplement for “Local connectome phenotypes predict social, health, and cognitive factors.” *Network Neuroscience*. 2(1): 86–105. https://doi.org/10.1162/netn_a_00031

Supplementary Table 1. Pairwise Pearson correlation matrix for 28 continuous HCP attributes. Asterisks indicate significant correlations after correcting for multiple comparisons (FDR < 0.05).

	Age (in years)	*Handedness*	Total Household Income	*Years of Education Completed*	*Body Mass Index*	*Mean Hematocrit Sample*	Diastolic Blood Pressure	*Systolic Blood Pressure*	Systolic-Diastolic Blood Pressure Ratio	Hemoglobin A1C	Pittsburgh Sleep Quality Index	*NIH Picture Sequence Memory Test*	NIH Dimensional Change Card Sort Test	NIH Flanker Inhibitory Control and Attention Test	*Penn Progressive Matrices: Number of Correct Responses*	*Penn Progressive Matrices: Total Skipped Items*	Penn Progressive Matrices: Median Reaction Time for Correct Responses	NIH Oral Reading Recognition Test	NIH Picture Vocabulary Test	NIH Toolbox Pattern Comparison Processing Speed Test	Delay Discounting: Area Under the Curve for Discounting of $200	*Delay Discounting: Area Under the Curve for Discounting of $40,000*	*Variable Short Penn Line Orientation: Total Number Correct*	Variable Short Penn Line Orientation: Median Reaction Time Divided by Expected Number of Clicks for Correct	Variable Short Penn Line Orientation: Total Positions Off for All Trials	Penn Word Memory Test: Total Number of Correct Responses	Penn Word Memory Test: Median Reaction Time for Correct Responses
Handedness	**0.11***																										
Total Household Income	**0.29***	0.02																									
Years of Education Completed	**0.15***	0.06	**0.40***																								
Body Mass Index	0.07	0	**-0.08***	**-0.13***																							
Mean Hematocrit Sample	**-0.15***	**-0.13***	0.02	0.06	0.04																						
Diastolic Blood Pressure	0.03	-0.02	0.02	-0.01	**0.32***	**0.16***																					
Systolic Blood Pressure	-0.08	-0.03	-0.02	-0.06	**0.38***	**0.20***	**0.70***																				
Systolic-Diastolic Blood Pressure Ratio	**-0.11***	0.01	-0.03	0	0	0	**-0.54***	**0.15***																			
Hemoglobin A1C	**0.12***	0.04	-0.07	**-0.09***	**0.17***	**-0.08***	**0.09***	**0.1***	0																		
Pittsburgh Sleep Quality Index	-0.03	-0.06	-0.07	-0.05	0.07	0	0.03	0.02	0	0.03																	
NIH Picture Sequence Memory Test	-0.04	0.01	**0.13***	**0.22***	**-0.11***	0	**-0.11***	**-0.13***	0.02	-0.03	0																
NIH Dimensional Change Card Sort Test	**-0.12***	0	**0.13***	**0.18***	**-0.12***	0.06	0	0.03	0.06	-0.01	0	**0.21***															
NIH Flanker Inhibitory Control and Attention Test	**-0.13***	-0.05	**0.09***	**0.12***	-0.04	0.07	0.03	0.04	0.02	0.03	0	**0.14***	**0.53***														
Penn Progressive Matrices: Number of Correct Responses	**-0.11***	**-0.07***	**0.16***	**0.32***	**-0.15***	**0.10***	-0.03	-0.02	0.05	**-0.08***	-0.06	**0.25***	**0.18***	**0.13***													
Penn Progressive Matrices: Total Skipped Items	**0.17***	**0.13**	**-0.07***	**-0.15***	**0.18***	-0.04	**0.08***	0.06	-0.01	**0.15***	**0.12***	**-0.17***	**-0.12***	-0.04	**-0.86***												
Penn Progressive Matrices: Median Reaction Time for	-0.01	-0.03	**0.09***	**0.24***	-0.07	**0.11***	0.02	0.01	0.01	-0.05	-0.06	**0.11***	0.01	-0.02	**0.76***	**-0.68***											

Correct Responses																											
NIH Oral Reading Recognition Test	0.02	-0.06	0.26*	0.46*	-0.16*	0.05	-0.02	-0.01	0.03	-0.10*	-0.10*	0.12*	0.22*	0.18*	0.39*	-0.30*	0.24*										
NIH Picture Vocabulary Test	0.12*	-0.08*	0.23*	0.47*	-0.11*	0.14*	-0.01	-0.02	0.01	-0.09*	-0.08*	0.13*	0.14*	0.18*	0.39*	-0.29*	0.21*	0.65*									
NIH Toolbox Pattern Comparison Processing Speed Test	-0.13*	-0.07	0.09*	0.13*	-0.06	0.04	0.01	0	0	-0.03	-0.03	0.18*	0.43*	0.41*	0.12*	-0.04	-0.07	0.15*	0.13*								
Delay Discounting: Area Under the Curve for Discounting of $200	0.02	-0.03	0.14*	0.22*	-0.10*	0.01	-0.05	-0.06	-0.01	-0.01	-0.12*	0.09*	0	0.03	0.21*	-0.12*	0.18*	0.22*	0.25*	0.01							
Delay Discounting: Area Under the Curve for Discounting of $40,000	0.01	0.01	0.14*	0.25*	-0.11*	0.02	-0.08*	-0.05	0.04	-0.05	-0.11*	0.03	0.03	0.05	0.17*	-0.10*	0.18*	0.23*	0.25*	0.03	0.70*						
Variable Short Penn Line Orientation: Total Number Correct	-0.09*	-0.08*	0.10*	0.20*	-0.13*	0.11*	-0.08*	-0.05	0.06	-0.05	0	0.17*	0.24*	0.18*	0.40*	-0.31*	0.27*	0.35*	0.31*	0.14*	0.16*	0.17*					
Variable Short Penn Line Orientation: Median Reaction Time Divided by Expected Number of Clicks for Correct	0.04	-0.01	-0.02	0.06	0	0.02	-0.01	-0.04	-0.03	-0.02	0.01	0.01	-0.10*	-0.16*	0.22*	-0.15*	0.39*	0.05	0.02	-0.15*	0.07	0.05	0.13*				
Variable Short Penn Line Orientation: Total Positions Off for All Trials	0.13*	0.11*	-0.10*	-0.14*	0.14*	-0.15*	0.09*	0.05	-0.08*	0.11*	0.02	-0.17*	-0.24*	-0.21*	-0.42*	0.43*	-0.30*	-0.39*	-0.35*	-0.14*	-0.16*	-0.19*	-0.92*	-0.13*			
Penn Word Memory Test: Total Number of Correct Responses	-0.03	-0.01	0.09*	0.21*	-0.11*	-0.07	-0.03	-0.09*	-0.01	0.03	-0.04	0.23*	0.13*	0.06	0.16*	-0.06	0.07*	0.24*	0.21*	0.10*	0.10*	0.11*	0.14*	0.08*	-0.12*		
Penn Word Memory Test: Median Reaction Time for Correct Responses	0.14*	0.04	-0.03	-0.01	0.03	0	0.02	0	-0.02	0	0.03	-0.12*	-0.17*	-0.19*	-0.07	0.11*	0.11*	-0.15*	-0.17*	-0.28*	-0.03	-0.05	-0.01	0.27*	0.02	-0.26*	
NIH List Sorting Working Memory Test	-0.04	-0.06	0.20*	0.24*	-0.06	0.05	-0.01	0	0.02	-0.09*	-0.01	0.32*	0.19*	0.15*	0.34*	-0.25*	0.14*	0.33*	0.32*	0.19*	0.11*	0.10*	0.26*	-0.01	-0.27*	0.11*	-0.13*
